# Peri-Typhlitis, with Abscess of Appendix Vermiformis; Operation and Recovery

**Published:** 1890-04

**Authors:** W. A. Ellison

**Affiliations:** Manchaca


For Daniel's Texas Medical Journal.

### PERITYPHLITIS WITH ABSCESS OF APPENDIX VERMIFORMIS: OPERATION AND RECOVERY.

BY W. A. ELLISON, M. D., MANCHACA.

ON January 3, 1890, I was called to see Tom Davis, aged 14 years, who, though delicate, had always had good health. I was told that the patient had been kicked by a horse, and on making an examination, I found the humerus fractured at the upper third. I had no trouble in resetting the bone, and left directions for him to keep quiet.

On the night of the 8th I was called in haste to see the patient again. I found him suffering with severe pain in the abdomen, but more severe in the region of the cæcum, pains of an intermittent character; and by placing the hand on the abdomen I could feel the bowels cramping beneath. On making inquiry, I found that the patient had eaten a considerable amount of pecans on the 5th, and that on the 6th he had had a spell of colic, though not very severe, though the abdomen had been rather tender ever since; I also learned that his bowels had not moved for three days. There was at this time, considerable tympanitis and great tenderness. My diagnosis was colic or enteralgia due to non-digestion of the pecans eaten.

I gave at once a dose of Hoffman's anodyne, arom. spt. amman, and chloroform, also ordered a copious injection of warm water and soap, which was used, but without any result. I had the injection repeated several times during the next twelve hours, but he had no fecal discharge.

I also gave Epsom salts, and some pills of aloin, podophyllin, and some other ingredients, but without any result. Pain was so severe that I was compelled to give morphine, but even then there was very little relief.

Thinking that if I could possibly get the bowels to move, the patient would get relief, I concluded to give croton oil. I gave the croton oil one half drop doses every half hour until three doses had been given. Fifteen minutes after giving the third and last dose, the bowels moved freely, discharge thin and watery and very offensive. Three more discharges during the next hour. Some few lumps of hard fecal matter. Patient much easier, and sleeping. Bowels moved several times during the next twenty-four hours, patient feeling much better, and I began to think that he was about well.

January 10th, found the patient resting tolerably well, but complaining of considerable soreness in the bowels. On making a close examination, I found great tenderness in the ileo-cæcal region; I could also feel a considerable enlargement. Temperature and pulse about normal. Every hour or two the patient would have a slight attack of the colicy pains.

I saw at once that the boy had typhlitis, but I considered it rather a mild case; gave morphine to produce complete ease, and ordered him kept very quiet, also directed that an anodyne liniment be applied every hour or two.

January 11. Patient about the same, had rested most of the night; bowels still very sore; could plainly see the enlargement in the right iliac region.

I told the father that I feared that the boy would have an abscess, and I pointed out the dangers to him. Applied tr. iodine to the enlargement; also directed that turpentine stupes be applied to the entire abdomen, as warm as the patient could bear them. Bowels moved twice during the night, very little appetite, slight fever.

January 12 to 18. Patient suffering most of the time, requiring a large amount of tr. opium to produce any rest, occasional rigors, slight fever all the time. Abdomen very tympanitic, and tender. So much so that I could not define the enlargement spoken of; could not detect any dullness on percussion; breathing difficult on account of distention of the abdomen.

January 20. I found the patient no better; so I requested that I should have consultation. About the 21st or 22nd (don't remember the exact date), Dr. R. M. Swearingen, of Austin, saw the case with me, and agreed with me in diagnosis and treatment, except that he suggested that I give more opium, and substitute small hot poultices for the turpentine stupes applied directly over the vermiform appendix. We both were of the opinion that we had an abscess to deal with, though on the account of the great distention we could not define the tumor, nor could we get any fluctuation or dullness on percussion.

Very little change for the next three or four days; of course patient getting weaker all the time, also suffering a great deal in spite of the opiate.

Dr. Swearingen saw the patient again with me on the 26th and 30th, on the latter date could distinctly feel and see the enlargement; also some dullness, but we were not certain whether there was fluctuation or not; bowels very tympanitic, not much fever, no appetite, bowels move every day.

February 1. We both saw the patient again; debated some time as to whether we would introduce an aspirating needle or not, but finally concluded to wait a day or two more. I stayed with the patient all night. He passed a very bad night indeed. and early next morning I sent for Dr. Swearingen again.

Dr. S. arrived at 2 o'clock p. m., and we proceeded at once to operate. After producing complete anesthesia with chloroform, Dr. S. at my request introduced a large aspirating needle, and as soon as the air was exhausted from the aspirator, it immediately filled with pus. Withdrawing the aspirator he made a free incision about three-fourths of an inch long, through which gushed an abundance of thick pus; some of it having a very strong smell of fecal matter.

February 3. Patient feeling much better; abscess has discharged at least one quart in the last twelve hours; dressed the wound with borated cotton.

February 4. Patient rested very badly through the night, notwithstanding we gave him a large quantity of laudanum. Discharged quite a quantity of feces through the abscess. Odor very offensive.

February 6. Patient improving, rests better; wants to eat. Abscess still discharging pus freely, also some fecal matter passing through abscess.

February 7th to 10th. Patient still improving; no fever; appetite good, but digestion bad; compelled to give opium to relieve the colic pains, which are very severe at times. Tympanitis disappearing. No fecal discharge through the abscess since the 6th; bowels have not moved naturally since the 2nd. Ordered an injection of warm water and glycerine into the rectum, which he retained an hour or two without any desire to go to stool. Repeated the injection and then introduced a tube into the rectum and let the water flow out again; water considerably stained with feces. This seemed to give him considerable relief. The rectum seems to have lost all sensibility. The patient has no desire whatever to go to stool, even after a copious injection; ordered an injection every six hours and then a half an hour afterwards to introduce the tube and let the water pour off, if there was still no desire to pass it. This seemed to give a great deal of relief.

February 13. Patient still improving; bowels moved freely to-day, still has severe pains at times, though not so often; no fever; appetite good.

February 20. Patient has been gradually improving every day, but has some fever to-day and feels very stretchy; pain in back and legs. I diagnosed *la grippe;* gave antifebrine and quinine; fever lasted two days and gradually wore off.

March 1. Patient improving rapidly; abscess is still discharging slightly, pus healthy; no fecal matter passing through the abscess since February 6. Still has light attacks of colic; has taken no opium in several days. Bowels move twice a day. He is taking no medicine except elix. bismuth and pepsin after each meal; good appetite. Patient took a buggy ride to-day and enjoyed it very much.

March 12. Still a slight discharge every day or two of pus from the abscess. Patient says he feels entirely well; walks around in the house. Has had no pain in ten days.

March 13. Patient and his mother think there was a slight discharge of feces through the abscess to-day; they say it looked and smelled like it. I am not certain whether the discharge was fecal or not; I did not see it; perhaps it was, but I can hardly think that there is still an opening into the gut, as there has been no dischage ot fecal matter for more than a month through the abscess until to-day.

March 16. Slight discharge of fecal matter through the abscess to-day. Patient says he feels entirely well, and gaining strength rapidly; appetite and digestion good; sleeps well; able to walk all over the yard.

March 26. The external opening in the abscess seems determined to heal. Have kept it open until now by keeping a cotton string inserted and by applying warm poultices. No fecal discharge from abscess in ten days; almost no pus discharging. I am disposed to think that the opening from the intestine into the abscess has closed, and if so, the patient will be entirely well in a short time.

I will here remark that the fracture of the humerus united all right, except a slight angle at the point of fracture, due to the loosening of the bandage from the rapid loss of flesh and the boy being so restless and in so much pain that it was impossible to keep the arm well confined.

I will also remark here that the patient was unable to void his urine for several days at the time, during the last week in January. I taught the father to introdnce a soft rubber catheter; and had the urine drawn several times a day. I attributed the bladder trouble to the opium taken.

